# Degradation of [Dha^7^]MC-LR by a Microcystin Degrading Bacterium Isolated from Lake Rotoiti, New Zealand

**DOI:** 10.1155/2013/596429

**Published:** 2013-06-27

**Authors:** Theerasak Somdee, Michelle Thunders, John Ruck, Isabelle Lys, Margaret Allison, Rachel Page

**Affiliations:** ^1^Faculty of Sciences, Khon Kaen University, Khon Kaen 40000, Thailand; ^2^Institute of Food, Nutrition and Human Health, College of Health, Massey University at Wellington, Private Bag 756, Wellington 6140, New Zealand; ^3^Faculty of Engineering, Health, Science and the Environment, Charles Darwin University, Darwin NT 0815, Australia

## Abstract

For the first time a microcystin-degrading bacterium (NV-3 isolate) has been isolated and characterized from a NZ lake. Cyanobacterial blooms in New Zealand (NZ) waters contain microcystin (MC) hepatotoxins at concentrations which are a risk to animal and human health. Degradation of MCs by naturally occurring bacteria is an attractive bioremediation option for removing MCs from drinking and recreational water sources. The NV-3 isolate was identified by 16S rRNA sequence analysis and found to have 100% nucleotide sequence homology with the *Sphingomonas* MC-degrading bacterial strain MD-1 from Japan. The NV-3 isolate (concentration of 1.0 × 10^8^ CFU/mL) at 30°C degraded a mixture of [Dha^7^]MC-LR and MC-LR (concentration 25 **μ**g/mL) at a maximum rate of 8.33 **μ**g/mL/day. The intermediate by-products of [Dha^7^]MC-LR degradation were detected and similar to MC-LR degradation by-products. The presence of three genes (*mlr*A, *mlr*B, and *mlr*C), that encode three enzymes involved in the degradation of MC-LR, were identified in the NV-3 isolate. This study confirmed that degradation of [Dha^7^]MC-LR by the *Sphingomonas* isolate NV-3 occurred by a similar mechanism previously described for MC-LR by *Sphingomonas* strain MJ-PV (ACM-3962). This has important implications for potential bioremediation of toxic blooms containing a variety of MCs in NZ waters.

## 1. Introduction

Toxic cyanobacterial blooms in eutrophic lakes, ponds, and reservoirs are a common occurrence around the world [1–3]. Cyanobacteria of the genera *Microcystis*, *Anabaena*, *Nostoc,* and *Planktothrix* produces a wide range of potent toxins, including a family of heptapeptide hepatotoxins, referred to as microcystins (MCs). Microcystins are the most frequently detected cyanobacterial toxins, which cause hepatotoxicity and tumor promotion in wild animals, livestock, and humans [2, 4, 5]. Epidemiological studies of primary liver cancer in China and the death of 56 patients during a dialysis treatment in Caruaru, Brazil triggered worldwide concern about toxicity of MCs [4, 5]. Approximately 75 variants of MCs have been identified and MC-L (leucine) R (arginine) is the most common variant of MC worldwide [1, 6–9]. Limited studies in New Zealand (NZ) have reported the occurrence of the [Dha^7^]MC-LR variant occurring with high frequency in NZ waters [3, 10, 11].

Microcystins are chemically stable over a wide range of temperature and pH, possibly as a result of their cyclic structure [12]. The toxins are also resistant to enzymatic hydrolysis by some general proteases, such as pepsin, trypsin, collagenase and chymotrypsin [13]. However, in the presence of natural microbial populations, degradation of MCs can take place. In 1994, Jones et al. successfully isolated from Australian river water *Sphingomonas* strain MJ-PV (ACM-3962), a single bacterial strain that utilized MC-LR as its sole source of carbon, and nitrogen needed for growth [14]. Further research led to the elucidation of MC-LR degradation pathways of the bacterial strain ACM-3962 [15]. Two intermediate degradation products were identified, suggesting that at least three intracellular hydrolytic enzymes were involved in the degradation of MC-LR. The first enzyme identified in the degradation pathway, a metalloprotease named microcystinase, cleaves the aromatic ring of MC-LR at the Arg-Adda peptide bond. This step yields a linearized MC-LR, which has a 160-fold reduction in toxic activity compared with the parent MC-LR. Next, a serine peptidase catalyzes the linearized MC-LR at the Ala-Leu peptide bond, producing a tetrapeptide. Finally, the third enzyme, another metalloprotease, cuts the peptide bonds randomly resulting in undetectable peptide fragments and amino acids [16]. Bourne et al. [16] further performed cloning and molecular characterization of four genes (*mlr*A, B, C, and D) that encode the three hydrolytic enzymes plus a putative oligopeptide transporter, all involved in the MC degradation metabolic pathway of the* Sphingomonas* strain ACM-3962.

Cyanobacterial blooms and MC production in NZ water bodies have been investigated by Wood [3] who detected MCs in 102 water samples from 54 different locations. The samples collected from Lake Rotoiti, Lake Hakanoa, Lake Horowhenua, and Lake Waitawa in April 2003 contained high levels of MCs, ranging from 0.02 *μ*g ml^−1^ to a maximum of 36,500 *μ*g ml^−1^. Wood [3] revealed the presence of [Dha^7^]MC-LR, MC-LR, MC-RR, MC-AR, MC-FR, MC-LA, MC-WR, MC-YR, and MC-LY from Lake Horowhenua, and MC-LR, MC-RR, MC-AR, MC-FR, MC-LA, MC-WR, and MC-YR from Lake Rotoiti [17]. Somdee [18] purified seven MC variants from lyophilized bloom samples (20 g each) of *Microcystis aeruginosa* collected from Lake Horowhenua in May 2005, with [Dha^7^]MC-LR, the major variant (purity of 93% and total yield of 51.84 mg), along with moderate amounts of MC-LR, MC-RR, MC-dMe-RR, MC-AR, MC-FR, and MC-YR. Contamination of drinking water sources by cyanotoxins [19] remains a serious threat to animal and human health and thus degradation of MCs by naturally occurring bacteria is an attractive bioremediation option for removing MCs from drinking and recreational water sources and supplies. The objectives of this research were to (a) isolate and identify natural aquatic bacteria from NZ water bodies capable of degrading MCs, (b) ascertain optimal biodegradation conditions for the identified NZ MC-degrading bacteria, and (c) determine the biodegradation pathway by the identified NZ MC-degrading bacteria for [Dha^7^]MC-LR, the most common MC variant in NZ waters.

## 2. Materials and Methods

### 2.1. Isolation of MC-Degrading Bacteria

Water samples taken from Lake Rotoiti, Lake Rotorua, Lake Rotoehu, and Lake Horowhenua, during blooms of *Microcystis aeruginosa, *were used as potential sources of MC-degrading bacteria. 10 mL samples of lake water were inoculated into 190 mL of a sterile mineral salts medium (MSM) broth containing [Dha^7^]MC-LR and MC-LR as the main food source for 5 days and incubated in the dark at 30°C in a shaking incubator (200 rpm). Five subcultures were established per water sample. MC-degrading bacteria were isolated by streaking serial dilutions of 10^−2^ to 10^−8^ onto peptone-yeast extract medium agar plates (PYEM) and incubated at 30°C. Morphologically distinct colonies on the plates were restreaked onto new plates for purity. A single colony of each bacterial isolate was grown on 5 mL PYEM broth overnight at 30°C in a shaking incubator (200 rpm). 1 mL of overnight culture was inoculated into 19 mL of fresh MSM broth containing MCs at a final concentration of 25 *μ*g mL^−1^ and incubated at 30°C in a shaking incubator (200 rpm) for 7 days. The MC degradation products were analysed individually for each isolate. A 1 mL sample was withdrawn at 1 day intervals, centrifuged (12000 rpm for 10 min), and the supernatant analyzed on HPLC (UV detector). Bacterial isolate NV-3 was selected for further characterisation and examination of biodegradative capacity. 

### 2.2. 16S rRNA Sequencing of NV-3 Isolate

Sequencing of 16S rRNA was carried out by ESR, New Zealand, using an ABI PRISM BigDye terminator DNA sequencing kit and analyzed on a model 3730XL ABI DNA sequencer (Applied Biosystems). The DNA sequences were compared with the GenBank, EMBL, and DJB prokaryote databases using the default settings of the Fasta3 alignment programme through the EBI server. 

### 2.3. Extraction of [Dha^7^]MC-LR and MC-LR for Biodegradation Experiments

A mix of [Dha^7^]MC-LR and MC-LR was considered ideal as a substrate for the biodegradation experiments (determination of optimal degradation conditions and examination of biodegradative by-products), as extracts rich in [Dha^7^]MC-LR have not previously been studied, and New Zealand natural bloom sources were deemed more appropriate than pure commercial forms of MC. Extraction and purification of [Dha^7^]MC-LR and MC-LR MCs from lyophilized bloom samples were performed as described by Somdee [18]. The MC variants were identified by HPLC and LC-MS/MS. 

### 2.4. Investigation of Effect of Temperature, Bacterial, and MC Concentration on Degradation of MCs by NV-3 Isolate

The pure bacterial isolate NV-3 was cultured in a PYEM broth for 36 h (late exponential growth phase determined from bacterial growth curve experiment) in a shaking incubator at 30°C and 200 rpm. The bacterium was centrifuged at 12,000 rpm for 5 min (4°C). The supernatant was decanted, and the pellet was resuspended in 0.05 M phosphate buffer, pH 7.0. This washing process was repeated three times. The final pellet was resuspended in 5 mL of sterile MSM broth, and this bacterial culture was used for examining the effect of temperature, bacterial, and MC concentrations on MC degradation by the bacterium isolate NV-3.

### 2.5. Temperature Experiments

 Sterile MSM broth was added to the prepared bacterial culture to adjust the concentration of the stock culture to give an optical density of 1.0 (OD_600_) and bacterial concentration of approximately 1.0 × 10^8^ CFU/mL. To establish the effect of temperature on MC degradation, 9 mL of the NV-3 bacterial culture was mixed with 1 mL of [Dha^7^]MC-LR and MC-LR (25 *μ*g/mL final concentration). The experiment was carried out in triplicate at 6 different temperatures, 10, 15, 20, 25, 30, and 35°C, in a shaking incubator at 200 rpm. The biodegradation of MCs was monitored for each experiment over a period of 28 days. An aliquot (1 mL) of the bacterial/cyanotoxin mix was withdrawn after 0, 1, 3, 5, 7, 10, 14, 21, and 28 days of incubation and centrifuged at 12,000 rpm for 10 min. The MC concentration was determined in the supernatant using HPLC.

### 2.6. Bacterial Concentration Experiments

Using the optimum temperature for MC degradation, experiments to determine the effect of bacterial concentration on MC degradation were carried out at different bacterial concentrations with a fixed MC concentration of 25 *μ*g/mL. Five bacterial concentrations were prepared OD_600_ = 0.1 (bacterial concentration approximately 7.9 × 10^6^ CFU/mL), OD_600_ = 0.3 (2.5 × 10^7^ CFU/mL), OD_600_ = 0.5 (4.9 × 10^7^ CFU/mL), OD_600_ = 1.0 (1.0 × 10^8^CFU/mL), and OD_600_ = 1.5 (1.45 × 10^8^ CFU/mL), using the prepared bacterial culture and adding sterile MSM broth until the required OD at 600 nm was obtained. A 9 mL sample of each bacterial concentration was mixed with 1 mL of [Dha^7^]MC-LR and MC-LR (25 *μ*g/mL final concentration). The experiments were carried out in a shaking incubator at the optimum temperature (30°C) and at 200 rpm. Progressive biodegradation of MCs was monitored over 3 days.

### 2.7. Substrate (Microcystin) Concentration Experiments

The optimum temperature and bacterial concentration were used to establish optimum MC concentration for bacterial degradation. 9 mL of the bacterial culture (OD_600_ = 1.0) was mixed with 1 mL of [Dha^7^]MC-LR and MC-LR, yielding final MC concentrations of 1, 10, 25, and 50 *μ*g/mL, and incubated in a shaking incubator at 30°C and 200 rpm. The progressive biodegradation of the MCs was monitored over 6 days.

### 2.8. Detection of MC-Degradation By-Products

The [Dha^7^]MC-LR and MC-LR variants extracted and purified from natural algal bloom were used in the biodegradation assays for detection of MC-degradation by-products by the NV-3 isolate. The pure NV-3 isolate was transferred to 19 mL of fresh MSM broth, containing MCs at a final concentration of 25 *μ*g mL^−1^ and incubated in a shaking incubator at 30°C and 200 rpm. Samples (1 mL) were taken every 6 h until 48 h, and the MC-degradation by-products were detected and analyzed using LC/MS-MS at Cawthron Institute, Nelson, New Zealand. 

### 2.9. Detection of Genes Encoding MC-Degrading Enzymes

The isolated bacterium (NV-3 isolate) was cultured in peptone-yeast extract broth for 36 h at 30°C and 200 rpm. Genomic DNA was extracted using the Wizard Genomic DNA Purification Kit (Promega) and quantified by a biophotometer (Eppendorf) with A260/280 ratio. PCR primers for amplification of *mlr*A gene are listed in Saito et al. [20] and for *mlr*B, *mlr*C, and *mlr*D genes are described in Ho et al. [21]. The PCR reactions were composed of 1 ng genomic DNA, 1 pmol of each primer, 1× PCR buffer (Invitrogen), 2 mM dNTPs (Invitrogen), 2.5 mM MgCl_2_ (Invitrogen), and 1.25 units of AmpliTaq Gold DNA polymerase (Applied Biosystems), giving a final volume of 20 *μ*L. The amplification was performed on a programmable thermal cycler (hybaid P×2 thermal cycler) with temperatures and times as previously described by Saito et al. [20] for mlrA and Ho et al. [21] for *mlrB*, C, and D. The PCR products were then purified using the Wizard SV gel and PCR clean-up system (Promega), and both strands were directly sequenced on an ABI-3730 automated sequencer (Applied Biosystems), at Allan Wilson Centre (AWC) Genome Sequencing Centre, Massey University, NZ. Assembled sequences were aligned using a ClustalW with MEGA 4.0 program (retrieved December 10, 2008, from http://www.megasoftware.net/) and then subjected to a nucleotide BLAST search. 

## 3. Results

Water samples obtained from Lake Rotoiti, Lake Rotoehu, Lake Rotorua, and Lake Horowhenua were plated on MSM broth with [Dha^7^]MC-LR and MC-LR as the sole carbon and nitrogen source. A total of 27 isolates of different types, shapes, and colors of colony were selected; however, only three isolates that were obtained from Lake Rotoiti, designated NV-1, NV-2 and NV-3, were truly able to break, down the MCs (data not shown). NV-3 isolate showed greatest degradation activity and was used for characterization and further experiments.

### 3.1. 16S rRNA Sequence of NV-3 Isolate

 The 16S rRNA sequence of the isolate NV-3 was determined and compared with the GenBank, EMBL, and DJB prokaryote databases. The databases revealed that the 16S rRNA sequences of the NV-3 isolate resemble the sequences of* Sphingomonas* strain MD-1 (AB110635), with 100% sequence homology for 1436 continuous nucleotides, and exhibits 98.5% homology with 16S rRNA of *Sphingomonas stygia* (AB025013). The NV-3 isolate was classified as a *Sphingomonas *sp., indistinguishable from the *Sphingomonas* strain MD1.

### 3.2. Effect of Temperature, Bacterial, and MC Concentration on Biodegradation

**Figure 1:**
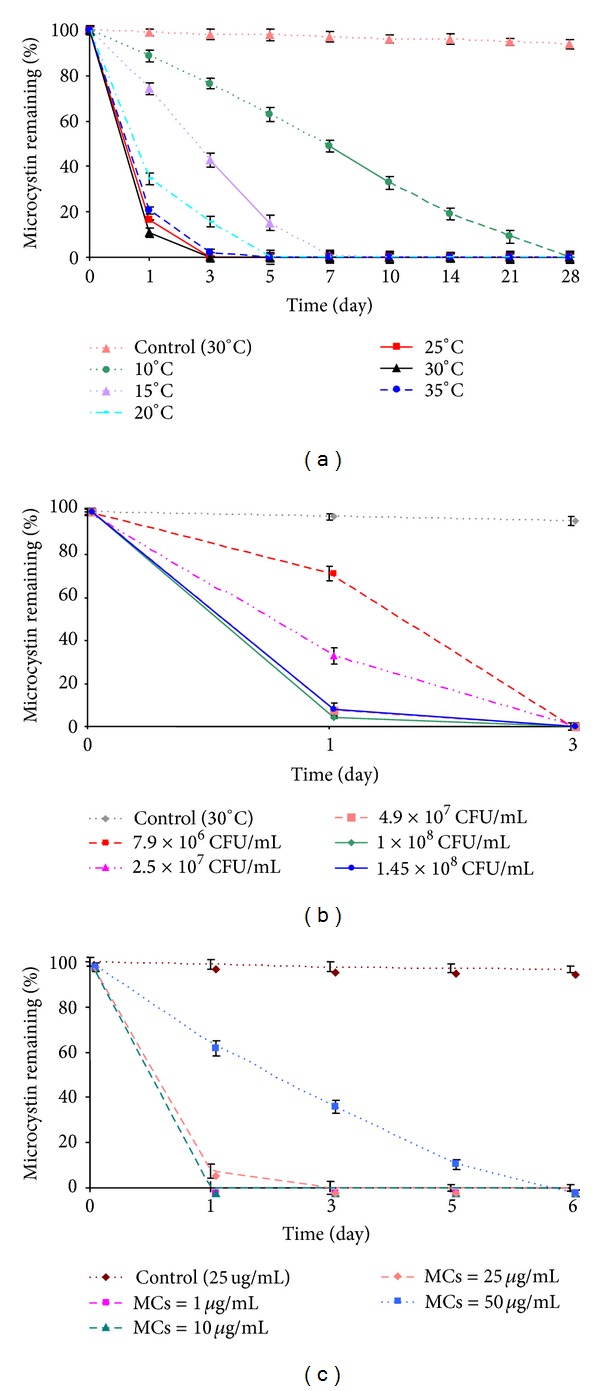
(a) Biodegradation of [Dha^7^]MC-LR and MC-LR with bacterial isolate NV-3 at temperatures of 10°, 15°, 20°, 25°, 30°, and 35°C; (b) varying rates of [Dha^7^]MC-LR and MC-LR biodegradations with varying bacterial concentrations. (c) [Dha^7^]MC-LR and MC-LR biodegradations at varying MC concentrations (1, 10, 25, and 50 *μ*g/mL).

The effect of temperature on the ability of NV-3 (1.0 × 10^8^ CFU/mL) to degrade 25 *μ*g/mL MCs was investigated. MC degradation by NV-3 began on day 1 under all temperatures tested (Figure 1(a)); however, the rate of MC degradation varied with temperature. The degradation rate was slowest at 10°C (0.89 *μ*g/mL/day) and steadily increased with increasing temperatures to 35°C (8.30 *μ*g/mL/day). MC concentrations rapidly decreased at temperatures from 20° to 35°C, with complete degradation occurring within 5 days (Figure 1(a)). However, at these temperatures a noticeable drop off in degradation rate occurred after the initial rapid phase. The highest degradation rate was reached with temperatures of 30°C (8.33 *μ*g/mL/day); however, the rate of degradation during the initial rapid phase was similar between temperatures of 25, 30, and 35°C and was calculated to be approximately 8.30 *μ*g/mL/day. These experiments demonstrated that the optimum temperature for NV-3 isolate biodegradation of [Dha^7^]MC-LR and MC-LR variants was 30°C.

The optimum bacterial concentration of NV-3 isolate for degradation of 25 *μ*g/mL MCs was then investigated at 30°C (being the optimum temperature for MC degradation by NV-3). Bacterial concentrations of 7.9 × 10^6^, 2.5 × 10^7^, 1.0 × 10^8^, and 1.45 × 10^8^ CFU/mL completely degraded the MCs within 3 days (Figure 1(b)). However, the degradation rate was slow with the lowest bacterial concentrations 7.9 × 10^6^ and 2.5 × 10^7^ CFU/mL. After one day of incubation with 7.9 × 10^6^ and 2.5 × 10^7^ CFU/mL NV-3 isolate, the amount of [Dha^7^]MC-LR remaining was 80% and 40%, respectively. By contrast, after incubation with the higher NV-3 isolate concentrations of 4.9 × 10^7^, 1.0 × 10^8^, and 1.45 × 10^8^ CFU/mL, less than 20% of [Dha^7^]MC-LR remained. The MC degradation rate in general increased with increasing bacterial concentration. At bacterial concentrations of 4.9 × 10^7^, 1.0 × 10^8^ and 1.45 × 10^8^ CFU/mL, the degradation rates were not significantly different with the same degradation rate of 8.33 *μ*g/mL/day. However, the degradation rate actually decreased slightly at the highest bacterial concentration of 1.45 × 10^8^ CFU/mL. These experiments demonstrated that the optimum bacterial concentration for NV-3 isolate biodegradation of [Dha^7^]MC-LR and MC-LR variants was 1.0 × 10^8^ CFU/mL. The effect of variation in MC concentration on NV-3 isolate degradation capability was also investigated at 30°C and NV-3 concentration 1.0 × 10^8^ CFU/mL. At low MC concentrations (1 and10 *μ*g/mL), the toxins were completely degraded in one day, whereas at higher concentrations of toxins (25 and 50 *μ*g/mL) degradation took longer, reaching undetectable levels by day 3 and 6 respectively (Figure 1(c)). The rate of degradation for concentrations of 25 and 50 *μ*g/mL was equal at 8.33 *μ*g/mL/day.

### 3.3. Detection of MC-Degrading By-Products

**Figure 2:**
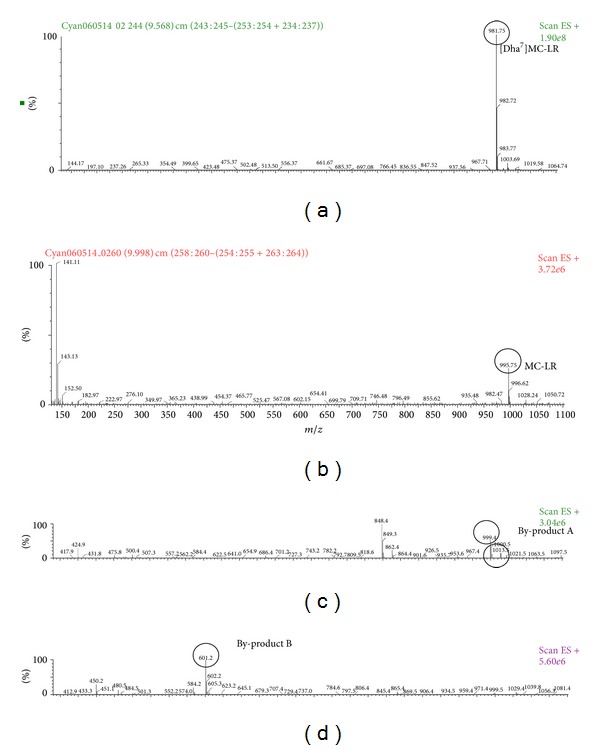
Visualisation of MS/MS spectrum data of degradation by-products of [Dha^7^]MC-LR and MC-LR in ESI^+^ with parent ion spectrum for MS-MS channels set up. (a) [Dha^7^]MC-LR at *m/z* 981.75, (b) MC-LR at *m/z* 995.75, (c) [Dha^7^]MC-LR degradation by-product A at *m/z* 999.4 and MC-LR degradation by product A at *m/z* 1013.5, (d) [Dha^7^]MC-LR degradation by-product B at *m/z* 601.2.

Biodegradation of MCs was induced using a mixture of [Dha^7^]MC-LR and MC-LR (25 *μ*g/mL final concentration) with cell suspensions of the isolate NV-3 (1.0 × 10^8^ CFU/mL). The toxin concentration began to decline, and two peaks, referred to as by-products A and B, were detected in a HPLC chromatogram. The peaks corresponding to the by-products increased gradually, while the peak corresponding to the parent toxins decreased. The by-products from microbial catabolism were further analyzed using LC/MS-MS. [Dha^7^]MC-LR is 14 mass units less than MC-LR due to the loss of a methyl group at Mdha, and the *m*/*z* at 981.75 and 995.75 confirmed that [Dha^7^]MC-LR, and MC-LR, respectively, were present in the biodegradation assay samples (Figures 2(a) and 2(b)). By-product A revealed the base peak at *m/z* 999 (Figure 2(c)) and was analogous to the linearized peptide of [Dha^7^]MC-LR (NH_2_-Adda-D-Glu-Dha-D-Ala-L-Leu-D-MeAsp-L-Arg-OH). The ion spectra of the prominent ion at *m/z* 848.4 (Figure 2(c)), which was from the loss of PhCH_2_CHOMe from the linearized peptide of [Dha^7^]MC-LR, confirmed the ring opening of [Dha^7^]MC-LR. The biodegradation by-product B revealed the base peak at *m/z* 601.2 (Figure 2(d)). The [M+H]^+^ ion 601.2 is the tetrapeptide of [Dha^7^]MC-LR (NH_2_-Adda-D-Glu-Dha-D-Ala-OH), 14 mass units less than the tetrapeptide of MC-LR microbial degradation reported in Bourne et al. [15]. 

### 3.4. Detection of MC-Degrading Genes

DNA fragments of the NV-3 isolate *mlr*A (720 bp), B (340 bp), C (590 bp), and D (600 bp) genes were PCR amplified and then directly sequenced. The DNA sequences were investigated using BLASTN search. The *mlr*A nucleotides (721 bp) exhibited a 99% DNA sequence similarity to the 807 base pair nucleotide sequence of the *mlr*A gene from *Sphingomonas* strain MD-1 (NCBI accession number AB114202). The *mlr*B337 bp revealed a 94% similarity to the 448 nucleotide base pair sequence of the *mlr*B gene from *Sphingopyxis* sp. LH21 (DQ423530), whereas the nucleotide sequence of *mlr*C (588 bp) and *mlr*D (597 bp) genes was 99% and 97% similar to the respective genes (with 666 bp and 671 bp) from *Sphingomonas* sp. ACM-3962 (AF411070 and AF411071). 

## 4. Discussion

The presence of microcystins (MCs) in freshwater is becoming an increasing problem and poses a potential threat to human health around the world. The MC toxin is well recognized as a stable and persistent compound; however, reports have shown that MCs are vulnerable to break down by indigenous bacteria established in natural water [2, 14, 22–25]. This is the first study to isolate and characterize indigenous MC-degrading bacteria in New Zealand waters. We have demonstrated that the bacterium isolate NV-3 from Lake Rotoiti is able to utilise [Dha^7^]MC-LR and MC-LR as a sole source of carbon and energy. On the basis of 16S rRNA sequences the isolate NV-3 is indistinguishable from the *Sphingomonas *strain MD-1 from Japan. 

The rate of MC degradation by any bacterial isolate, including NV-3, relies mainly on the incubation temperature as well as bacterial concentration. Biodegradation of MCs by the bacterium occurred under a wide range of temperatures between 10°C and 35°C. At 10°C, the biodegradation was very slow and identical to that of the MC degrading-bacterium *Sphingopyxis *strain LH21, isolated from Australia, suggesting that it would similarly not be able to degrade MCs at 4°C [21]. The ability of bacteria to degrade toxins in low temperatures is relevant to the degradation of toxins in winter, for example, when the water temperature is low. For NV-3, increases in water temperature from 15°C to 30°C were associated with an increase in biodegradation rate; however, temperatures higher than the optimum temperature (30°C) for NV-3 bacterial growth resulted in slightly decreased degradative ability. It is likely that at high temperatures, NV-3 bacterial cells are unable to produce MC-degrading enzymes, or the cells might be inactive or growing slowly. The highest rate of MC-degradation by the isolate NV-3 was achieved at 30°C, a similar temperature observed for maximum MC-degradation by the *Sphingomonas* strain Y2, isolated from Lake Suwa, Japan [26], and for *Sphingopyxis *strain TT25 isolated from Australian waters, the highest rate was achieved at 25°C [27]. At the optimum temperature of bacterial growth, the bacterial metabolism is very active, producing a lot of enzymes responsible for MC degradation. By contrast, at temperatures that are lower or higher than that for optimum growth, bacterial metabolism and the production of biodegradation enzymes are presumably less active, slowing the rate of MC degradation.

Biodegradation of toxins is also dependent on bacterial concentration. In this study, the degradation rate increased with increasing bacterial cell concentration; however, concentrations above 1.0 × 10^8^ CFU/mL resulted in a decrease in degradative ability. This decrease might be due to competition or inhibition between the cells at very high cell numbers, and, therefore, the optimum cell density is also crucial for toxin degradation. In this study, the optimum cell density of the bacterium isolate NV-3, required for MC degradation experiments, was between 4.9 × 10^7^ to 1.0 × 10^8^ CFU/mL and the minimum number of cells required was approximately 7.9 × 10^6^ CFU/mL. It is interesting that at the minimum cell density, MCs were completely degraded within 3 days, the same length of time taken with higher concentrations of bacteria. This confirms the ability of the bacterium isolate NV-3 to utilise the toxins as their own food and to multiply itself to a cell density sufficient to completely degrade the MCs. The effect of toxin concentrations on MC degradation by NV-3 was also examined. In this study, at the optimum temperature (30°C) and bacterial concentration (1.0 × 10^8^ CFU/mL), the biodegradation rate increased with increasing concentrations of the toxins (the mixture of [Dha^7^]MC-LR, and MC-LR). The degradation rate by the bacterial isolate was identical (i.e., 8.33 *μ*g/mL/day) for moderate to high concentrations of the toxins (25 *μ*g/mL to 50 *μ*g/mL). It is possible that as the sole carbon and nitrogen sources for bacterial growth, high concentrations of MCs were beneficial to growth of the isolated bacteria. However, the apparent inhibitory effects of very high toxin concentrations (i.e., >50 *μ*g/mL) and the limit to the amount of MCs that NV-3 can degrade need to be further investigated, as the effects on bacterial growth and metabolism are unknown. 

**Figure 3:**
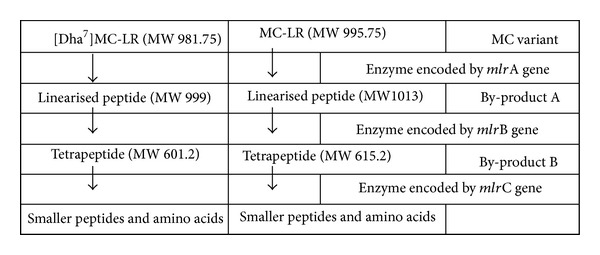
Putative pathway for [Dha^7^]MC-LR degradation by *Sphingomonas* isolate NV-3 based on published data regarding MC-LR degradation pathway [15, 16]. Refer to Figure 2 for visualisation of degradation by-products. MW: molecular weight.

Biodegradation studies of MCs have been mainly focused on the MC-LR variant since it is found worldwide and possesses high toxicity (LD_50_ (ip) = 50 *μ*g/kg in mice) [1, 8, 9, 28]. Other analogues of MCs such as MC-RR, YR, LW, and LF have also been examined [13, 26, 29–35]. In New Zealand waters it appears that [Dha^7^]MC-LR is in greater quantity than MC-LR [3, 10, 11]. In this study, in addition to MC-LR, [Dha^7^]MC-LR was used as a substrate to characterize the biodegradation pathway of [Dha^7^]MC-LR using the bacteriumisolate NV-3. Previous studies have shown that at least three hydrolytic enzymes are involved in degradation of MC-LR [15, 16]. Our study has shown that the by-products A and B of [Dha^7^]MC-LR (Figures 2(c) and 2(d)) are degradation products equivalent to what happens to MC-LR (Figure 3) in an enzymatic pathway identical to that described by Bourne et al. [15]. It is important to note that the MC-degradation by-products, namely linearized peptides, tetrapeptides and peptide fragments are significantly less toxic than the parent molecules of MCs. Bourne et al. [15] demonstrated that the toxicity of the linearized peptides of MC-LR is reduced 160-fold compared with the parent compound, while other studies have demonstrated that tetrapeptide and amino acids are nontoxic [36]. These findings strongly indicate that microbial degradation is a potentially safe and natural treatment for removing MCs from water [37]. 

## 5. Conclusion 

In summary, (1) bacteria were found in New Zealand lakes with the ability to degrade [Dha^7^]MC-LR and MC-LR as the sole carbon and nitrogen sources; (2) 16S RNA of the MC-degrading bacteria isolated were indistinguishable from a previously identified bacterium MD-1 which was located in Japan; (3) optimal NV-3 degradation of 25 *μ*g/mL [Dha^7^]MC-LR and MC-LR prepared form a natural algal bloom, occurred at 30°C with bacterial concentration of 1.0 × 10^8^ CFU/mL, and(4) the by-products A and B from biodegradation of [Dha^7^]MC-LR and the detection of *mlr*A, *mlr*B, *mlr*C, and *mlr*D genes in NV-3 genome indicate that degradation of [Dha^7^]MC-LR is via a similar mechanism for degradation of MC-LR as described by Bourne et al. [15, 16]. This study has demonstrated that microcystin-degrading bacteria are present in New Zealand water bodies, and that these bacteria could be used potentially on a larger scale for removing microcystins from water.

### 5.1. Nucleotide Sequence Accession Numbers

The four gene sequences *mlr*A,* mlr*B,* mlr*C, and *mlr*D obtained for *Sphingomonas *NV-3 isolate have been deposited in GenBank under accession numbers JN256930, JN256929, JN256928, and JN256927, respectively.
